# Effect of pemafibrate on liver enzymes and shear wave velocity in non-alcoholic fatty liver disease patients

**DOI:** 10.3389/fmed.2023.1073025

**Published:** 2023-02-07

**Authors:** Ryosuke Sugimoto, Motoh Iwasa, Akiko Eguchi, Yasuyuki Tamai, Ryuta Shigefuku, Naoto Fujiwara, Hideaki Tanaka, Yoshinao Kobayashi, Jiro Ikoma, Masahiko Kaito, Hayato Nakagawa

**Affiliations:** ^1^Department of Gastroenterology and Hepatology, Graduate School of Medicine, Mie University, Tsu, Japan; ^2^Center for Physical and Mental Health, Graduate School of Medicine, Mie University, Tsu, Japan; ^3^Mie Shokakinaika, Tsu, Japan

**Keywords:** NAFLD, dyslipidemia, pemafibrate, liver function, liver fibrosis

## Abstract

**Background/Aims:**

Pemafibrate is a selective peroxisome proliferator-activated receptor α modulator that improves serum alanine aminotransferase (ALT) in dyslipidemia patients. Pemafibrate was reported to reduce ALT in non-alcoholic fatty liver disease (NAFLD) patients, but efficacy was not clearly elucidated due to the small size of previous study populations. Therefore, we explored pemafibrate efficacy in NAFLD patients.

**Methods:**

We retrospectively evaluated pemafibrate efficacy on liver enzymes (*n* = 132) and liver shear wave velocity (SWV, *n* = 51) in NAFLD patients who had taken pemafibrate for at least 24 weeks.

**Results:**

Patient ALT levels were decreased from 81.0 IU/L at baseline to 48.0 IU/L at week 24 (*P* < 0.0001). Serum levels of aspartate aminotransferase (AST), γ-glutamyl transpeptidase (γ-GTP) and triglyceride (TG) were significantly decreased, and high-density lipoprotein cholesterol and platelet count were significantly increased, with no change in body weight being observed. Study participant SWV values decreased from 1.45 m/s at baseline to 1.32 m/s at week 48 (*P* < 0.001). Older age (*P* = 0.035) and serum TG levels (*P* = 0.048) were significantly associated with normalized ALT. Changes in AST, ALT, γ-GTP and body weight were significantly correlated with change in SWV.

**Conclusion:**

Pemafibrate significantly improves liver function, serum TG and liver stiffness in NAFLD patients. Pemafibrate is a promising therapeutic agent for NAFLD and may be a candidate for NAFLD patients with elevated TG.

## Introduction

Non-alcoholic fatty liver disease (NAFLD) is the most prevalent chronic liver disease with an approximate 25% global morbidity rate (1). If left untreated, NAFLD can progress to liver cirrhosis and hepatocellular carcinoma (2). Furthermore, NAFLD is associated with extrahepatic complications, particularly cardiovascular disease and certain cancers (3, 4). Although weight loss and lifestyle modification behaviors are the first therapeutic approach to prevent NAFLD, or prevent the progression of NAFLD, most patients do not easily adhere to this approach (5, 6). Currently, there exists a dearth of pharmacological recommendations tailored to the treatment of NAFLD (5, 6).

Pemafibrate is a selective peroxisome proliferator-activated receptor (PPAR) α modulator family that was designed to have a higher PPARα agonistic activity and selectivity than existing PPARα agonists (such as fibrates) (7). The efficacy of pemafibrate on reducing cardiovascular events had been examined in a large-scale clinical study (8, 9) and then, pemafibrate is currently marketed for the treatment of hypertriglyceridemia since 2018 only in Japan. PPARα has anti-inflammatory properties through transrepression of pro-inflammatory target genes, such as activator protein (AP)-1 and nuclear factor (NF)-κB (10).

Corresponding to PPARα effect, pemafibrate has the potential to be beneficial to NAFLD patients. Recent human and animal studies suggest that PPARα activation by pemafibrate promotes lipid turnover with simultaneous inducement of triglyceride (TG) hydrolysis, fatty acid β-oxidation, TG synthesis and TG re-esterification in the liver (11–13). Additionally, pemafibrate improved liver fibrosis by reducing collagen 1α1 mRNA levels along with a decrease in the levels of pro-inflammatory mRNA (12). Indeed, pemafibrate induced a significant reduction in alanine aminotransferase (ALT) and γ-glutamyl transpeptidase (γ-GTP) levels in NAFLD patients (11, 14–21). However, the efficacy of pemafibrate with respect to NAFLD was not addressed in these earlier studies due to cohort size. In addition, the efficacy of pemafibrate with respect to the amelioration of hepatic fibrosis has yet to be clearly demonstrated. Remarkable recent advances in imaging technology have allowed clinicians to monitor and assess changes in liver tissue using ultrasound imaging techniques, such as vibration controlled transient elastography (FibroScan) and shear wave elastography (SWE) (22).

In this study, our aim was to retrospectively evaluate the efficacy of pemafibrate on liver enzymes and liver shear wave velocity (SWV) in NAFLD patients.

## Materials and methods

### Study population

This is a retrospective, observational study. Prior to the initiation of the study, the study protocol was reviewed and approved by the clinical research ethics review committee of Mie University hospital (Approval No. H2022-012). Since this was a retrospective medical record survey, it was exempt from written or oral consent; however, we released information about this research so patients could opt out of having their data used.

We selected patients who had taken pemafibrate from June 2018 to December 2021 and obtained information about medical history and medication (e.g., age, gender, body weight, comorbidities, and treatments for type 2 diabetes, hypertension, and dyslipidemia). The inclusion criteria were thus: (1) fatty liver diagnosed by abdominal ultrasonography, such as increased liver echogenicity, liver-kidney contrast, and poor visualization of deep hepatic parenchyma, (2) dyslipidemia treated with pemafibrate (0.1 mg twice a day), (3) alcohol consumption < 30 g/day in males and < 20 g/day in females. Exclusion criteria were as follows: (1) severe chronic kidney disease [serum creatinine (Cr) > 2.5 mg/dl], (2) cirrhosis, hepatocellular carcinoma, gallstones or other chronic liver diseases, such as viral infection and autoimmune hepatitis, (3) patients who stopped pemafibrate within 24 weeks, (4) patients who were newly prescribed sodium-glucose cotransporter type 2 inhibitors, glucagon-like peptide-1 agonists and/or pioglitazone.

### Intervention and outcomes

All patients were given weight-loss counseling 12 weeks before receiving pemafibrate. Patients received pemafibrate (0.1 mg twice daily) orally for at least 24 weeks and visited an outpatient clinic every 2–8 weeks. Any drugs administered prior to starting pemafibrate were continued during the observation period. Biochemical parameters, including hepatic function, lipid metabolism and renal function, were measured at baseline and again at 24 weeks. The SWV values in m/s, as determined using an ARIETTA S70 (Hitachi Aloka Medical, Ltd, Japan), were measured at baseline and again at 48 weeks. Patients were divided into four groups according to SWV values of <1.34 m/s, ≥1.34 to <1.55 m/s (significant fibrosis), ≥1.55 to <1.80 m/s to (severe fibrosis) and ≥ 1.80 m/s (liver cirrhosis), respectively (23). The primary end-point analysis was change in patient serum ALT levels from baseline. Secondary end-point analyses included change in aspartate aminotransferase (AST), γ-GTP, TG, low-density lipoprotein cholesterol (LDL-C), high-density lipoprotein cholesterol (HDL-C), Cr, platelet (Plt) count, fibrosis-4 (FIB-4), body weight (kg) and SMV. The FIB-4 values were calculated using the formula: age (years) × AST (IU/L)/(Plt [10^9^/L] × [ALT [IU/L])1/2) (24).

### Statistical analysis

Data are represented as mean values with standard deviation (SD). All changes between baseline and week 24 or 48 were analyzed using two-tailed paired t-tests. The correlation analysis was carried out using Spearman’s correlation analysis. Normalized or 30% decrease in laboratory tests were evaluated using McNemar test. The associations of normalized ALT (<30 IU/L), 30% decrease in ALT and 30% decrease in SWV (25) after pemafibrate treatment with baseline characteristics were assessed using logistic regression model. In the model, continuous variables were log_2_-transformed to mitigate the skewed distribution. Younger age (<40 years old) was defined based on the 1st quartile in this cohort. Variables with *P* < 0.10 in univariable analyses were included in a multivariable analysis for adjustment. A *P*-value <0.05 was considered statistically significant. Statistical analyses were performed using Prism (GraphPad Software, Inc., San Diego, CA, USA) and SPSS Statistics 27.0 (IBM, Armonk, NY, USA) and R software version 4.1.0^1^.

## Results

### Baseline characteristics of study patients

The baseline characteristics of the 132 NAFLD patients who had taken pemafibrate are shown in Table 1. The baseline values of ALT, γ-GTP, TG and HDL-C were 81.0 ± 50.0 IU/L, 89.8 ± 80.8 IU/L, 235.2 ± 165.4 mg/dL and 50.0 ± 14.4 mg/dL, respectively. FIB-4 index at baseline was 1.24 ± 1.14, indicating that the majority of patients would be clinically categorized as having low or intermediate risk of advanced fibrosis (26). Serial SWV values were available for 51 out of the 132 NAFLD patients. Within these 51 NAFLD patients, the baseline values of ALT and γ-GTP were 90.7 ± 63.1 IU/L and 99.7 ± 94.9 IU/L, respectively. FIB-4 index at baseline was 1.25 ± 1.16, indicating that the majority of patients would be clinically categorized as low or intermediate risk of having advanced fibrosis (Table 2).

**TABLE 1:** Characteristics of patients with NAFLD at baseline and after 24 weeks of pemafibrate treatment.

*n* = 132	Baseline	24 weeks	*P*-value
Male/female	100/32		
Age (years)	48.5±14.0		
Body weight (kg)	76.5±13.8	76.2±14.1	ns
Hypertension (%)	10		
Diabetes mellitus (%)	11		
AST (IU/L)	48.7±28.4	36.4±19.6	<0.0001
ALT (IU/L)	81.0 ± 50.0	48.0 ± 33.0	<0.0001
γ-GTP (IU/L)	89.8 ± 80.8	49.9 ± 51.6	<0.0001
TG (mg/dL)	235.2 ± 165.4	127.0 ± 72.5	<0.0001
LDL-C (mg/dL)	131.7 ± 28.1	114.0 ± 28.7	<0.0001
HDL-C (mg/dL)	50.0 ± 14.4	52.4 ± 12.9	<0.0001
Cr (mg/dL)	0.82 ± 0.17	0.86 ± 0.17	<0.0001
Plt (×10^4^/μL)	25.2 ± 5.6	27.3 ± 6.2	<0.0001
FIB-4	1.24 ± 1.14	1.13 ± 0.94	<0.001

NAFLD, non-alcoholic fatty liver disease; AST, aspartate aminotransferase; ALT, alanine aminotransferase; γ-GTP, γ-glutamyl transpeptidase; TG, triglyceride; LDL-C, low-density lipoprotein cholesterol; HDL-C, high-density lipoprotein cholesterol; Cr, creatinine; Plt, platelet; FIB-4, fibrosis-4.

**TABLE 2:** Characteristics of patients measured liver stiffness using SWE at baseline and after 48 weeks of pemafibrate treatment.

*n* = 51	Baseline	48 weeks	*P*-value
Male/female	35/16		
Age (years)	49.4±14.3		
Body weight (kg)	76.1±12.3	75.4±12.8	ns
Hypertension (%)	10		
Diabetes mellitus (%)	8		
AST (IU/L)	53.3 ± 35.6	31.7 ± 18.2	<0.0001
ALT (IU/L)	90.7 ± 63.1	40.3 ± 40.0	<0.0001
γ-GTP (IU/L)	99.7 ± 94.9	44.3 ± 46.8	<0.0001
TG (mg/dL)	227.6 ± 152.8	123.2 ± 67.8	<0.0001
LDL-C (mg/dL)	133.6 ± 25.8	114.5 ± 25.4	<0.0001
HDL-C (mg/dL)	50.1 ± 14.0	53.6 ± 13.6	<0.05
Cr (mg/dL)	0.82 ± 0.17	0.85 ± 0.18	<0.001
Plt (×10^4^/μL)	25.4 ± 5.5	27.1 ± 6.5	<0.005
FIB-4	1.25 ± 1.16	1.06 ± 0.74	<0.05

SWE, shear wave elastography; AST, aspartate aminotransferase; ALT, alanine aminotransferase; γ-GTP, γ-glutamyl transpeptidase; TG, triglyceride; LDL-C, low-density lipoprotein cholesterol; HDL-C, high-density lipoprotein cholesterol; Cr, creatinine; Plt, platelet; FIB-4, fibrosis-4.

### Change in laboratory and clinical data after pemafibrate treatment

**FIGURE 1:**
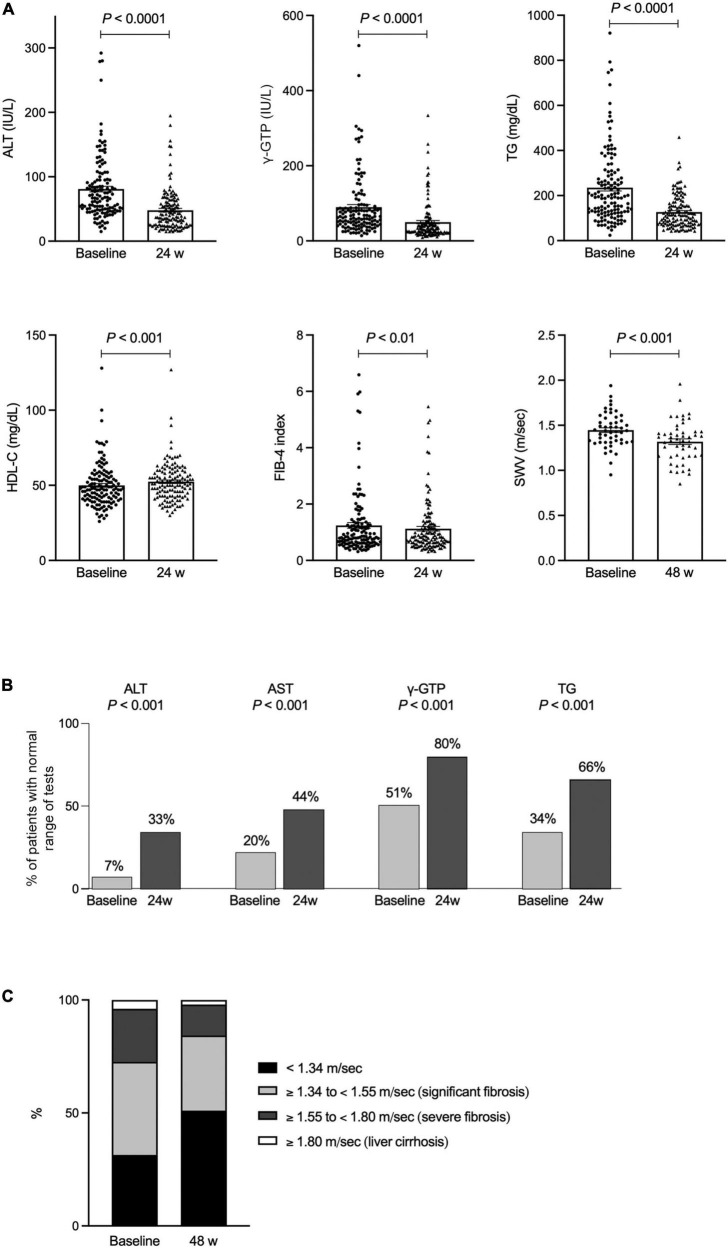
**(A)** The changes in ALT, γ-GTP, TG, HDL-C, FIB-4 index and SWV at baseline and 24 or 48 weeks of treatment with pemafibrate. **(B)** Normalized ALT as well as AST, γ-GTP, and TG after 24-week treatment with pemafibrate compared to the baseline. **(C)** The changes in fibrosis stage estimated SWV at baseline and 48 weeks of treatment with pemafibrate. ALT, alanine aminotransferase; γ-GTP, γ-glutamyl transpeptidase; TG, triglyceride; HDL-C, high-density lipoprotein cholesterol; FIB-4, fibrosis-4; SWV, shear wave velocity; AST, aspartate aminotransferase.

We determined that no adverse events, including liver or renal injury, were reported during the treatment period. The changes in general data from baseline to week 24 are summarized in Table 1 and the changes in ALT, γ-GTP, TG and HDL-C at baseline and week 24 are shown in Figure 1A. Patient ALT levels decreased from 81.0 IU/L at baseline to 48.0 IU/L at week 24 (*P* < 0.0001). Patient serum levels of AST, γ-GTP and TG were significantly decreased and HDL-C, Cr and Plt count were significantly increased at the 24-week timepoint. The FIB-4 index, used as a marker of liver fibrosis, decreased from 1.24 at baseline to 1.13 at week 24 (*P* < 0.01; Figure 1A). Normalized ALT as well as AST, γ-GTP and TG were more frequently observed after 24-week treatment with pemafibrate compared to the baseline (Figure 1B). There was no change in patient body weight between baseline and week 24. The SWV measurement decreased from 1.45 m/s at baseline to 1.32 m/s at week 48 (*P* < 0.001; Figure 1A). Overall, pemafibrate treatment was associated with an improvement in the fibrosis stage estimated by SWV compared with the pretreatment stage (Figure 1C). As with the week 24 timepoint, there was no observed change in body weight between baseline and week 48 (75.8 ± 13.9 kg).

### Influence of pemafibrate on creatinine

Since serum Cr levels were increased after pemafibrate treatment, we investigated the factors associated with increasing serum Cr levels. We found no difference between the baseline clinical characteristics and laboratory variables in the increased Cr group versus the non-increased group (Supplementary Table 1). In addition, only the baseline Cr level < 0.9 mg/dL (odds ratio [OR], 2.7; 95% confidence interval [CI], 1.26–5.72; *P* = 0.011) was significantly associated with increased Cr in univariate logistic regression analysis.

### Parameters associated with changes in ALT

**FIGURE 2:**
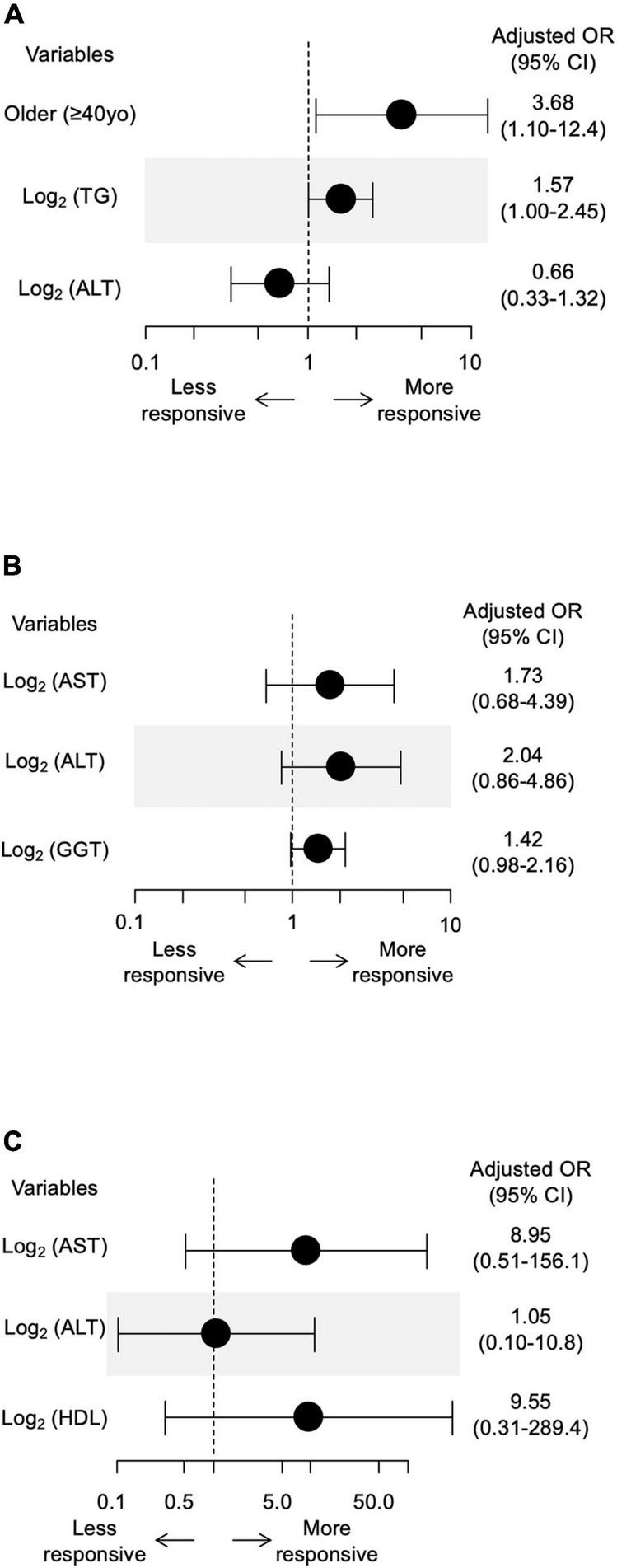
**(A)** The associations of normalized ALT after pemafibrate treatment with baseline age, TG and ALT. **(B)** The associations between 30% decrease in ALT after pemafibrate treatment with baseline AST, ALT, and γ-GTP. **(C)** The associations between 30% decrease in SWV after pemafibrate treatment with baseline AST, ALT, and HDL-C. TG, triglyceride; ALT, alanine aminotransferase; SWV, shear wave velocity; AST, aspartate aminotransferase; γ-GTP, γ-glutamyl transpeptidase; HDL-C, high-density lipoprotein cholesterol.

Among patients with elevated ALT at baseline (*n* = 123), 37 (30%) patients achieved normalized ALT 24 weeks after pemafibrate initiation. Older age (OR, 4.2; 95% CI, 1.36–13.0; *P* = 0.013), serum TG level (OR, 1.72; 95% CI, 0.93–3.21; *P* = 0.086), and ALT (OR, 0.39; 95% CI, 0.16–0.96; *P* = 0.041) at baseline were associated with normalized ALT (Table 3). Even after adjusting for these confounding factors, older age (adjusted OR, 3.68; 95% CI, 1.10–12.4; *P* = 0.035) and serum TG level (OR, 1.57; 95% CI, 1.00–2.45; *P* = 0.048) were significantly associated with normalized ALT (Figure 2A). Baseline levels of AST (OR, 3.28; 95% CI, 1.78–6.05; *P* < 0.001), ALT (OR, 3.18; 95% CI, 1.80–5.62; *P* < 0.001) and γ-GTP (OR, 1.61; 95% CI, 1.10–2.35; *P* = 0.013) were associated with a 30% decrease in ALT (Table 3). We observed no significant association between a 30% decrease in ALT and other baseline parameters after adjusting for these confounding factors (Figure 2B).

**TABLE 3:** The associations of normalized ALT, 30% decrease in ALT, and 30% decrease in SWV after pemafibrate treatment with the baseline characteristics.

Variables	Normalized ALT			30% decrease in ALT			30% decrease in SWV		
	OR	(95% CI)	*P* values	OR	(95% CI)	*P* values	OR	(95% CI)	*P* values
Male sex	0.71	(0.29–1.74)	0.46	1.07	(0.48–2.40)	0.86	0.19	(0.03–1.30)	0.09
Age (<40 years)	0.24	(0.8–0.74)	0.013	1.18	(0.53–2.64)	0.68	2.37	(0.34–16.4)	0.38
Diabetes	1.76	(0.52–6.00)	0.36	1.97	(0.58–6.64)	0.27	NA	NA	NA
Hypertension	0.86	(0.21–3.44)	0.83	0.85	(0.27–2.67)	0.77	NA	NA	NA
AST	0.74	(0.31–1.74)	0.49	3.28	(1.78–6.05)	<0.001	11.4	(1.85–70.0)	0.009
ALT	0.39	(0.16–0.96)	0.041	3.18	(1.80–5.62)	<0.001	4.1	(1.18–14.3)	0.027
γ-GTP	1.46	(0.83–2.56)	0.19	1.61	(1.10–2.35)	0.013	1.22	(0.48–3.12)	0.67
TG	1.72	(0.92–3.21)	0.086	1.29	(0.89–1.87)	0.19	1.04	(0.35–3.11)	0.94
LDL-C	1.46	(0.27–7.97)	0.66	2.38	(0.82–6.91)	0.11	0.52	(0.02–14.0)	0.70
HDL-C	1.65	(0.37–7.37)	0.51	0.75	(0.30–1.90)	0.54	18.6	(1.22–282.8)	0.036
Plt	0.75	(0.14–4.04)	0.74	0.55	(0.19–1.57)	0.27	0.53	(0.13–94.7)	0.45
FIB-4	1.53	(0.85–2.74)	0.16	1.33	(0.0–1.97)	0.15	1.74	(0.70–4.35)	0.24

SWV, shear wave velocity; OR, odds ratio; CI, confidence interval; AST, aspartate aminotransferase; ALT, alanine aminotransferase; γ-GTP, γ-glutamyl transpeptidase; TG, triglyceride; LDL-C, low-density lipoprotein cholesterol; HDL-C, high-density lipoprotein cholesterol; Cr, creatinine; Plt, platelet; FIB-4, fibrosis-4.

### Parameters associated with changes in SWV

**FIGURE 3:**
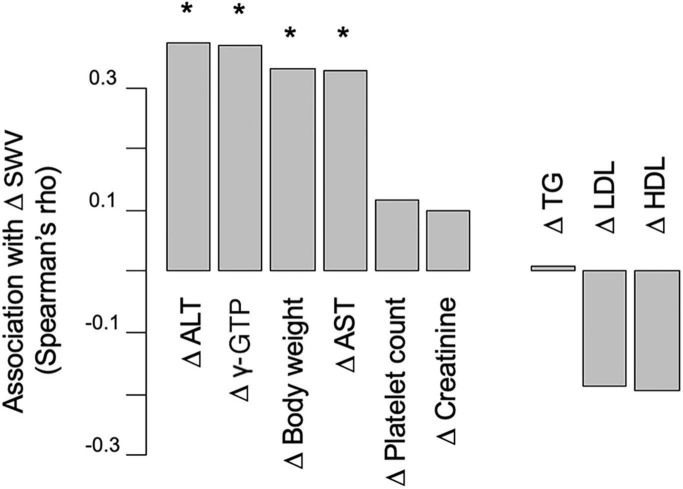
The association between change in SWV and changes in laboratory variables. SWV, shear wave velocity; AST, aspartate aminotransferase; ALT, alanine aminotransferase; γ-GTP, γ-glutamyl transpeptidase; TG, triglyceride; HDL-C, high-density lipoprotein cholesterol. *P < 0.05.

Baseline levels of AST (OR, 11.4; 95% CI, 1.85–70.0; *P* = 0.009), ALT (OR, 4.1; 95% CI, 1.18–14.3; *P* = 0.027) and HDL-C (OR, 18.6; 95% CI, 1.22–282.8; *P* = 0.036) were associated with a 30% decrease in SWV (Table 3). We observed no significant association between a 30% decrease in SWV and other baseline parameters after adjusting for these confounding factors (Figure 2C). We further investigated the association between change in SWV (SWV at week 48 minus SWV at baseline) and changes in clinical parameters. Changes in AST, ALT, γ-GTP and body weight were significantly correlated with change in SWV (*P* < 0.05; Figure 3).

## Discussion

In the presented study, a relatively large number of NAFLD patients taking pemafibrate saw significantly decreased serum liver enzymes such as AST, ALT and γ-GTP. We also found serum lipids, such as TG and HDL-C, to be significantly improved when NAFLD patients were treated with pemafibrate. These results are consistent with previous reports (14–16) (11, 17–21). Notably, the change in liver enzymes was accompanied with a significant improvement in liver stiffness, as measured by SWE.

We defined the primary endpoint in this study as change in serum ALT levels from baseline. In general, the reduction in serum ALT levels is a predictor of histological inflammation amelioration in patients with biopsy-proven NASH (27). In addition, Argo et al. reported that the presence of histological inflammation is the only predictor of fibrosis progression at follow-up biopsy (28). These results suggest that a reduction in serum ALT is a proxy index for histological improvement, including liver inflammation and fibrosis. In the present study, NAFLD patients undergoing pemafibrate therapy saw dramatically reduced ALT levels without a change in body weight. There exists the possibility that the effect of pemafibrate on liver function is independent of patient weight loss. Thus, a pemafibrate-associated reduction in serum ALT may be a beneficial treatment for patients with NAFLD/NASH. In addition, the predictive factor in this study related to the effect of pemafibrate was baseline serum TG levels. Therefore, pemafibrate may be suitable for NAFLD patients with high TG levels, although further study is required to validate the results.

For the first time, our study revealed that pemafibrate treatment led to a decrease in liver stiffness when assessed using SWE, however Nakajima et al. reported that pemafibrate showed a significant reduction in liver stiffness using another instrument, magnetic resonance elastography, in a double-blind, phase 2 study in patients with NAFLD (11). We demonstrated that the change in liver stiffness was accompanied with a significant improvement in blood liver fibrosis markers such as Plt count and FIB-4 index, which was consistent with previous reports (11, 15, 17, 18, 21). The significant reduction in liver stiffness and fibrosis markers suggests an overall improvement in liver fibrosis, indicating that pemafibrate may have another advantage in treating patients with liver fibrosis, since liver fibrosis is associated with an increased risk of developing liver-related events and is predictive in NAFLD patient prognosis (29). Considering that SWE-assessed liver stiffness is correlated with liver inflammation and hepatic steatosis (30, 31), and is positively correlated with changes in AST, ALT, γ-GTP, body weight and SWV in our study, our results of decreased liver stiffness may partially reflect a pemafibrate-related improvement in liver inflammation and hepatic steatosis. To be sure, further research is required to confirm the histological improvement in detail, including in patients with more advanced liver fibrosis.

We found that pemafibrate slightly increased serum Cr levels during the treatment period, which has also been reported in other studies involving the administration of fibrates (8, 32). It has been argued that this increased Cr effect is benign because it is not associated with a reduction in glomerular filtration rate estimated by inulin clearance (32) and is essentially reversible after cessation of fibrates (33). It may be caused by an overproduction of Cr (34) although interference in the generation of vasodilatory prostaglandins has been proposed as an explanatory mechanism (35). Overall, reports show a very positive safety profile for pemafibrate, even in patients with chronic kidney disease (36). Indeed, a relatively high Cr level (≥0.9 mg/dL) was not associated with increased Cr after pemafibrate treatment in our patients with NAFLD.

Our study did have some limitations insofar as it was a retrospective, observational study without a control group and involved multi-sample blood collections. In the present study, histopathological evaluation of the liver was not performed. A prospective study, including histopathological evaluation, should be performed in the future.

In conclusion, our study included a relatively large number of patients, and our results are consistent with previous reports. According to existing evidence, which includes our study, pemafibrate can undoubtedly ameliorate liver dysfunction assessed by AST, ALT and γ-GTP levels in NAFLD patients after 24 weeks of administration. Further, pemafibrate also improves liver stiffness after more than 48 weeks of treatment. No remarkable adverse effects were reported in these studies.

## Data availability statement

The original contributions presented in this study are included in the article/Supplementary material, further inquiries can be directed to the corresponding author.

## Ethics statement

The study protocol was reviewed and approved by the Clinical Research Ethics Review Committee of Mie University Hospital (Approval No. H2022-012). The patients/participants provided their written informed consent to participate in this study.

## Author contributions

MI contributed to the conception and design of the study. RSu, YT, RSh, HT, YK JI, MK, and HN organized the database. AE and NF performed the statistical analysis. RSu and MI wrote the first draft of the manuscript. All authors contributed to manuscript revision, read, and approved the submitted version.

## Footnotes

1
www.r-project.org

## References

[1] Younossi Z Koenig A Abdelatif D Fazel Y Henry L Wymer M. Global epidemiology of nonalcoholic fatty liver disease-meta-analytic assessment of prevalence, incidence, and outcomes. *Hepatology.* (2016) 64:73–84. 10.1002/hep.28431 26707365

[2] Farrell G Larter C. Nonalcoholic fatty liver disease: from steatosis to cirrhosis. *Hepatology.* (2006) 43(Suppl. 1):S99–112. 10.1002/hep.20973 16447287

[3] Lonardo A Nascimbeni F Mantovani A Targher G. Hypertension, diabetes, atherosclerosis and nash: cause or consequence? *J Hepatol.* (2018) 68:335–52. 10.1016/j.jhep.2017.09.021 29122390

[4] Bjorkstrom K Widman L Hagstrom H. Risk of hepatic and extrahepatic cancer in nafld: a population-based cohort study. *Liver Int.* (2022) 42:820–8. 10.1111/liv.15195 35152526 PMC9306866

[5] Tokushige K Ikejima K Ono M Eguchi Y Kamada Y Itoh Y Evidence-based clinical practice guidelines for nonalcoholic fatty liver disease/nonalcoholic steatohepatitis 2020. *J Gastroenterol.* (2021) 56:951–63. 10.1007/s00535-021-01796-x 34533632 PMC8531062

[6] Tokushige K Ikejima K Ono M Eguchi Y Kamada Y Itoh Y Evidence-based clinical practice guidelines for nonalcoholic fatty liver disease/nonalcoholic steatohepatitis 2020. *Hepatol Res.* (2021) 51:1013–25. 10.1111/hepr.13688 34533266

[7] Fruchart J. Selective peroxisome proliferator-activated receptor alpha modulators (SPPARMALPHA): the next generation of peroxisome proliferator-activated receptor alpha-agonists. *Cardiovasc Diabetol.* (2013) 12:82. 10.1186/1475-2840-12-82 23721199 PMC3682868

[8] Ishibashi S Arai H Yokote K Araki E Suganami H Yamashita S Efficacy and safety of pemafibrate (K-877), a selective peroxisome proliferator-activated receptor alpha modulator, in patients with dyslipidemia: results from a 24-week, randomized, double blind, active-controlled, phase 3 trial. *J Clin Lipidol.* (2018) 12:173–84. 10.1016/j.jacl.2017.10.006 29203092

[9] Pradhan A Paynter N Everett B Glynn R Amarenco P Elam M Rationale and design of the pemafibrate to reduce cardiovascular outcomes by reducing triglycerides in patients with diabetes (prominent) study. *Am Heart J.* (2018) 206:80–93. 10.1016/j.ahj.2018.09.011 30342298

[10] Delerive P De Bosscher K Besnard S Vanden Berghe W Peters J Gonzalez F Peroxisome proliferator-activated receptor alpha negatively regulates the vascular inflammatory gene response by negative cross-talk with transcription factors Nf-Kappab and Ap-1. *J Biol Chem.* (1999) 274:32048–54. 10.1074/jbc.274.45.32048 10542237

[11] Nakajima A Eguchi Y Yoneda M Imajo K Tamaki N Suganami H Randomised clinical trial: pemafibrate, a novel selective peroxisome proliferator-activated receptor alpha modulator (spparmalpha), versus placebo in patients with non-alcoholic fatty liver disease. *Aliment Pharmacol Ther.* (2021) 54:1263–77. 10.1111/apt.16596 34528723 PMC9292296

[12] Honda Y Kessoku T Ogawa Y Tomeno W Imajo K Fujita K Pemafibrate, a Novel selective peroxisome proliferator-activated receptor alpha modulator, improves the pathogenesis in a rodent model of nonalcoholic steatohepatitis. *Sci Rep.* (2017) 7:42477. 10.1038/srep42477 28195199 PMC5307366

[13] Sasaki Y Asahiyama M Tanaka T Yamamoto S Murakami K Kamiya W Pemafibrate, a selective pparalpha modulator, prevents non-alcoholic steatohepatitis development without reducing the hepatic triglyceride content. *Sci Rep.* (2020) 10:7818. 10.1038/s41598-020-64902-8 32385406 PMC7210999

[14] Yanai H Katsuyama H Hakoshima M. Effects of a novel selective peroxisome proliferator-activated receptor alpha modulator, pemafibrate, on metabolic parameters: a retrospective longitudinal study. *Biomedicines.* (2022) 10:401. 10.3390/biomedicines10020401 35203610 PMC8962310

[15] Ikeda S Sugihara T Kihara T Matsuki Y Nagahara T Takata T Pemafibrate ameliorates liver dysfunction and fatty liver in patients with non-alcoholic fatty liver disease with hypertriglyceridemia: a retrospective study with the outcome after a mid-term follow-up. *Diagnostics.* (2021) 11:2316. 10.3390/diagnostics11122316 34943553 PMC8700575

[16] Hatanaka T Kosone T Saito N Takakusagi S Tojima H Naganuma A Effect of 48-Week pemafibrate on non-alcoholic fatty liver disease with hypertriglyceridemia, as evaluated by the fibroscan-aspartate aminotransferase score. *JGH Open.* (2021) 5:1183–9. 10.1002/jgh3.12650 34622006 PMC8485409

[17] Shinozaki S Tahara T Lefor A Ogura M. Pemafibrate improves hepatic inflammation, function and fibrosis in patients with non-alcoholic fatty liver disease: a one-year observational study. *Clin Exp Hepatol.* (2021) 7:172–7. 10.5114/ceh.2021.106864 34295984 PMC8284174

[18] Hatanaka T Kakizaki S Saito N Nakano Y Nakano S Hazama Y Impact of pemafibrate in patients with hypertriglyceridemia and metabolic dysfunction-associated fatty liver disease pathologically diagnosed with non-alcoholic steatohepatitis: a retrospective, single-arm study. *Intern Med.* (2021) 60:2167–74. 10.2169/internalmedicine.6574-20 33612679 PMC8355409

[19] Shinozaki S Tahara T Lefor A Ogura M. Pemafibrate decreases markers of hepatic inflammation in patients with non-alcoholic fatty liver disease. *Clin Exp Hepatol.* (2020) 6:270–4. 10.5114/ceh.2020.99528 33145434 PMC7592096

[20] Seko Y Yamaguchi K Umemura A Yano K Takahashi A Okishio S Effect of pemafibrate on fatty acid levels and liver enzymes in non-alcoholic fatty liver disease patients with dyslipidemia: a single-arm, pilot study. *Hepatol Res.* (2020) 50:1328–36. 10.1111/hepr.13571 32926754

[21] Ikeda S Sugihara T Hoshino Y Matsuki Y Nagahara T Okano J Pemafibrate dramatically ameliorated the values of liver function tests and fibrosis marker in patients with non-alcoholic fatty liver disease. *Yonago Acta Med.* (2020) 63:188–97. 10.33160/yam.2020.08.009 32884438 PMC7435118

[22] Takahashi H Ono N Eguchi Y Eguchi T Kitajima Y Kawaguchi Y Evaluation of acoustic radiation force impulse elastography for fibrosis staging of chronic liver disease: a pilot study. *Liver Int.* (2010) 30:538–45. 10.1111/j.1478-3231.2009.02130.x 19874490

[23] Friedrich-Rust M Nierhoff J Lupsor M Sporea I Fierbinteanu-Braticevici C Strobel D Performance of acoustic radiation force impulse imaging for the staging of liver fibrosis: a pooled meta-analysis. *J Viral Hepat.* (2012) 19:e212–9. 10.1111/j.1365-2893.2011.01537.x 22239521

[24] Vallet-Pichard A Mallet V Nalpas B Verkarre V Nalpas A Dhalluin-Venier V Fib-4: an inexpensive and accurate marker of fibrosis in hcv infection. comparison with liver biopsy and fibrotest. *Hepatology.* (2007) 46:32–6. 10.1002/hep.21669 17567829

[25] Hezode C Castera L Roudot-Thoraval F Bouvier-Alias M Rosa I Roulot D Liver stiffness diminishes with antiviral response in chronic hepatitis C. *Aliment Pharmacol Ther.* (2011) 34:656–63. 10.1111/j.1365-2036.2011.04765.x 21752038

[26] Shah A Lydecker A Murray K Tetri B Contos M Sanyal A Comparison of noninvasive markers of fibrosis in patients with nonalcoholic fatty liver disease. *Clin Gastroenterol Hepatol.* (2009) 7:1104–12. 10.1016/j.cgh.2009.05.033 19523535 PMC3079239

[27] Seko Y Sumida Y Tanaka S Mori K Taketani H Ishiba H Serum alanine aminotransferase predicts the histological course of non-alcoholic steatohepatitis in Japanese patients. *Hepatol Res.* (2015) 45:E53–61. 10.1111/hepr.12456 25429984

[28] Argo C Northup P Al-Osaimi A Caldwell S. Systematic review of risk factors for fibrosis progression in non-alcoholic steatohepatitis. *J Hepatol.* (2009) 51:371–9. 10.1016/j.jhep.2009.03.019 19501928

[29] Ekstedt M Hagstrom H Nasr P Fredrikson M Stal P Kechagias S Fibrosis stage is the strongest predictor for disease-specific mortality in nafld after up to 33 years of follow-up. *Hepatology.* (2015) 61:1547–54. 10.1002/hep.27368 25125077

[30] Jang J Lee E Seo J Kim Y Kim S Cho Y Two-dimensional shear-wave elastography and us attenuation imaging for nonalcoholic steatohepatitis diagnosis: a cross-sectional, multicenter study. *Radiology.* (2022) 305:118–26. 10.1148/radiol.220220 35727151

[31] Lee D Cho E Bae J Lee J Yu S Kim H Accuracy of two-dimensional shear wave elastography and attenuation imaging for evaluation of patients with nonalcoholic steatohepatitis. *Clin Gastroenterol Hepatol.* (2021) 19:797–805.e7. 10.1016/j.cgh.2020.05.034 32450363

[32] Ansquer J Dalton R Causse E Crimet D Le Malicot K Foucher C. Effect of fenofibrate on kidney function: a 6-week randomized crossover trial in healthy people. *Am J Kidney Dis.* (2008) 51:904–13. 10.1053/j.ajkd.2008.01.014 18501783

[33] Mychaleckyj J Craven T Nayak U Buse J Crouse J Elam M Reversibility of fenofibrate therapy-induced renal function impairment in accord type 2 diabetic participants. *Diabetes Care.* (2012) 35:1008–14. 10.2337/dc11-1811 22432114 PMC3329840

[34] Hottelart C El Esper N Rose F Achard J Fournier A. Fenofibrate increases creatininemia by increasing metabolic production of creatinine. *Nephron.* (2002) 92:536–41. 10.1159/000064083 12372935

[35] Chen Y Quilley J. Fenofibrate treatment of diabetic rats reduces nitrosative stress, renal cyclooxygenase-2 expression, and enhanced renal prostaglandin release. *J Pharmacol Exp Ther.* (2008) 324:658–63. 10.1124/jpet.107.129197 17993607

[36] Yokote K Yamashita S Arai H Araki E Suganami H Ishibashi S Long-term efficacy and safety of pemafibrate, a novel selective peroxisome proliferator-activated receptor-alpha modulator (Spparmalpha), in dyslipidemic patients with renal impairment. *Int J Mol Sci.* (2019) 20:706. 10.3390/ijms20030706 30736366 PMC6386904

