# Supplementary material for: Effect of pemafibrate on liver enzymes and shear wave velocity in non-alcoholic fatty liver disease patients
Source: Front Med (Lausanne). 2023 Feb 7;10:1073025. doi: 10.3389/fmed.2023.1073025 (PMC9941328; doi:10.3389/fmed.2023.1073025)
Supplement: Supplementary file 1 [file Table_1.DOCX]

**Supplementary Table 1** Characteristics of patients with NAFLD in the increased and non-increased group of creatinine after pemafibrate treatment

|  | Cr increased group (n=86) | Cr non-increased group  (n=46) | p-value |
| --- | --- | --- | --- |
| male/female | 67 / 19 | 33 / 13 | 0.5589 |
| Age (years) | 47.9 ± 13.5 | 49.7 ± 14.6 | 0.5958 |
| Body weight (kg) | 76.1 ± 13.7 | 77.4 ± 13.8 | 0.4476 |
| Hypertension | 8 (9.3%) | 5 (10.9%) | 0.9123 |
| Diabetes mellitus | 9 (10.5%) | 5 (10.9%) | 0.9466 |
| AST (IU/L) | 49.2 ± 30.8 | 47.7 ± 23.0 | 0.6812 |
| ALT (IU/L) | 85.4 ± 57.0 | 72.7 ± 30.9 | 0.4871 |
| γ-GTP (IU/L) | 88.3 ± 72.3 | 92.5 ± 94.0 | 0.5877 |
| TG (mg/dL) | 235.0 ± 167.5 | 235.6 ± 159.6 | 0.8898 |
| LDL-C (mg/dL) | 131.3 ± 25.3 | 132.3 ± 32.5 | 0.9600 |
| HDL-C (mg/dL) | 50.5 ± 15.1 | 49.0 ± 13.0 | 0.6362 |
| Cr (mg/dL) | 0.81 ± 0.15 | 0.86 ± 0.20 | 0.0817 |
| Plt (×10^4^/μL) | 24.7 ± 5.3 | 26.3 ± 6.0 | 0.1804 |
| FIB-4 index | 1.20 ± 1.07 | 1.31 ± 1.26 | 0.9866 |

NAFLD, nonalcoholic fatty liver disease; AST, aspartate aminotransferase; ALT, alanine aminotransferase; γ‐GTP, γ‐glutamyl transpeptidase; TG, triglyceride; LDL-C, low-density lipoprotein cholesterol; HDL-C, high-density lipoprotein cholesterol; Cr, creatinine; Plt, platelet; FIB-4, fibrosis‐4
